# Somatic mutations in intracranial arteriovenous malformations

**DOI:** 10.1371/journal.pone.0226852

**Published:** 2019-12-31

**Authors:** Jeremy A. Goss, August Y. Huang, Edward Smith, Dennis J. Konczyk, Patrick J. Smits, Christopher L. Sudduth, Christopher Stapleton, Aman Patel, Sanda Alexandrescu, Matthew L. Warman, Arin K. Greene

**Affiliations:** 1 Department of Plastic & Oral Surgery, Boston Children’s Hospital, Harvard Medical School, Boston, MA, United States of America; 2 Department of Pathology, Boston Children’s Hospital, Harvard Medical School, Boston, MA, United States of America; 3 Department of Neurosurgery, Boston Children’s Hospital, Harvard Medical School, Boston, MA, United States of America; 4 Department of Neurosurgery, Massachusetts General Hospital, Harvard Medical School, Boston, MA, United States of America; 5 Department of Orthopedic Surgery, Boston Children’s Hospital, Harvard Medical School, Boston, MA, United States of America; Beth Israel Deaconess Medical Center, UNITED STATES

## Abstract

**Background:**

Intracranial arteriovenous malformation (AVM) is a common cause of primary intracerebral hemorrhage in young adults. Lesions typically are sporadic and contain somatic mutations in *KRAS* or *BRAF*. The purpose of this study was to identify somatic mutations in a cohort of participants with brain AVM and to determine if any genotype-phenotype associations exist.

**Methods:**

Human brain AVM specimens (n = 16) were collected during a clinically-indicated procedure and subjected to multiplex targeted sequencing using molecular inversion probe (MIP-seq) for mutations in *KRAS*, *BRAF*, *HRAS*, *NRAS*, and *MAP2K1*. Endothelial cells (ECs) were separated from non-ECs by immune-affinity purification. Droplet digital PCR (ddPCR) was used to confirm mutations and to screen for mutations that may have been missed by MIP-seq. Patient and AVM characteristics were recorded.

**Results:**

We detected somatic mutations in 10 of 16 specimens (63%). Eight had *KRAS* mutations [G12D (n = 5), G12V (n = 3)] and two had *BRAF* mutations [V600E (n = 1), Q636X (n = 1)]. We found no difference in age, sex, presenting symptom, AVM location, or AVM size between patients with a confirmed mutation and those without. Nor did we observe differences in these features between patients with *KRAS* or *BRAF* mutations. However, two patients with *BRAF* mutations presented at an older age than other study participants.

**Conclusions:**

Somatic mutations in *KRAS* and, less commonly in *BRAF*, are found in many but not all intracranial AVM samples. Currently, there are no obvious genotype-phenotype correlations that can be used to predict whether a somatic mutation will be detected and, if so, which gene will be mutated.

## Introduction

Arteriovenous malformation (AVM) is a fast-flow vascular anomaly consisting of connections between arteries and veins through a nidus or fistula instead of a normal capillary bed [1]. Although AVMs can affect any anatomic location, intracranial AVM is the most common type, affecting approximately 10/100,000 persons [2]. Cerebral AVMs are a common cause of primary intracerebral hemorrhage in young adults [3]. The lesions can rupture resulting in hemorrhagic stroke. Rare familial brain AVMs associated with germline mutations can occur with hereditary hemorrhagic telangiectasia (*ENG*, *ACVRL1*, *SMAD4*, *GDF*) or capillary malformation-arteriovenous malformation (*RASA1* or *EPHB4*) [4–8]. However, almost all intracranial AVMs are non-hereditary and sporadic. Somatic *KRAS* mutations have been identified in the majority of brain AVM specimens [9–11]. The purpose of this study was to identify novel mutations in intracranial AVMs and to determine whether a genotype-phenotype correlation exists.

## Materials and methods

The Committee on Clinical Investigation at Boston Children’s Hospital approved this study. Written informed consent was obtained from all patients and/or their guardians when applicable prior to inclusion in the study. AVM specimens were collected during a clinically-indicated procedure and were flash-frozen and stored at -80°C. Specimens included in the study were limited to pial-type AVMs. DNA was extracted from frozen tissue and peripheral blood using DNeasy Blood & Tissue Kit (Qiagen). Two AVM specimens were also processed to separate endothelial cells (ECs) from other cell types (non-ECs) as previously described prior to DNA extraction [1].

Samples were subjected to high-coverage multiplex targeted sequencing using molecular inversion probe (MIP-seq) to search for somatic mutations. Genomic DNA for the protein-coding sequences of *KRAS*, *BRAF*, *HRAS*, *NRAS*, and *MAP2K1* was enriched by hybridization to probes containing an 8-nucleotide barcode that uniquely identified individual MIPs. Specimen DNA quality and quantity were analyzed by a DNA library control check, and inadequate specimens were excluded from MIP-seq. For the remaining specimens, MIP capture and sequencing were performed as previously described [12]: raw reads were aligned to the reference human genome sequence (GRCh37) and PCR duplicates were removed with Picard. MIP reads with the same molecular barcode then were collapsed to generate the consensus read, and candidate somatic mutations were called when the mutant allele was supported by two or more consensus reads. Somatic variants were considered true positives by MIP-seq if they were supported by 2 independent reads and the mutant allelic frequency (MAF) was greater than 1%. Mutations previously reported in the COSMIC cancer database [13] were given priority for independent validation by ddPCR. The MIP sequences used in this study are listed in S1 Table.

We developed ddPCR assays, as previously described [1,12], to confirm mutations suggested by MIP-seq, and we used ddPCR to determine if these mutations were present in specimens that tested negative by MIP-seq. After analyzing a minimum of 1,500 droplets per specimen, we considered ddPCR assays with a MAF >1% to be true positives. The presence or absence of a somatic mutation, or the gene affected by the somatic mutation, then were tested for statistically significant (*p* < 0.05) associations with patient age at presentation, sex, presenting symptom, AVM size and location, as well as patient radiologic and histopathologic findings.

Statistical analysis was performed using IBM SPSS Statistics for Windows version 26.0 (IBM Corp., Armonk, NY). Sex was compared between groups using Fisher’s exact test. Presenting symptom and location were compared using the Pearson chi-square test. Median age and size were compared using the nonparametric Mann-Whitney U test.

## Results

After collapsing smMIPs with the same barcode, we achieved > 150-fold coverage for 85% of the protein coding sequences for *KRAS*, *BRAF*, *HRAS*, *NRAS*, and *MAP2K1*. Because *KRAS* codon p.12G and *BRAF* codon p.600V somatic mutations have been linked to brain AVMs [9–11], we obtained > 500-fold coverage for these regions of each gene in all 16 specimens, and >5,000-fold coverage in 15/16 AVM specimens. Thus, for most AVM specimens we could detect a MAF as low as 0.04% at these codons. Ten of the 16 specimens (63%) had a likely somatic disease-causing mutation (Table 1). Eight specimens contained *KRAS* mutations, which included p.G12D (n = 5) and p.G12V (n = 3). Two specimens contained *BRAF* mutations: p.V600E and p.Q636X. In the two AVM samples from which ECs were immune-affinity separated from non-ECs (Participant 1 and Participant 5, Table 1), the MAF was more abundant in the EC fraction (1.7% and 22.4%, respectively) compared to the non-EC fraction (0% for each). Mutant alleles were not detected in blood samples from two participants who had somatic mutations detected in their AVM.

**Table 1:** Intracranial arteriovenous malformation (AVM) specimens and genetic results.

Participant	Age	Sex	Symptom	Location	Size (cm^2^)	Mutation	MAF (%)	
							MIPs	ddPCR
1	11	F	Headache	Frontal (Right)	7.5	*KRAS* p.G12D	-	1.0
2	11	F	Headache	Occipital (Right)	2.6	ND		
3	13	F	Seizure	Parietal (Right)	1.8	*KRAS* p.G12V	2.8	1.3
4	13	F	Seizure	Parietal-occipital (Right)	42.0	*KRAS* p.G12D	2.0	1.6
5	14	F	Headache	Parietal-occipital (Right)	4.6	*KRAS* p.G12V	-	1.8
6	14	M	Headache	Temporal (Right)	12.0	ND		
7	15	M	Headache	Temporal (Right)	7.8	*KRAS* p.G12D	2.7	1.5
8	15	F	Headache	Occipital (Right)	6.0	ND		
9	16	M	Seizure	Parietal (Right)	12.5	*KRAS* p.G12D	0.2	3.5
10	16	F	Hemiplegia	Temporal (Left)	6.0	ND		
11	17	M	Seizure	Frontal (Right)	12.0	ND		
12	19	M	Headache	Temporal (Right)	18.0	*KRAS* p.G12D	0.6	2.7
13	20	M	Headache	Temporoparietal (Right)	8.4	ND		
14	21	M	Seizure	Frontal (Left)	2.4	*KRAS* p.G12V	3.8	2.8
15	28	F	Headache	Occipital (Right)	5.0	*BRAF* p.V600E	-	2.2
16	46	F	Headache	Occipital (Right)	8.0	*BRAF* p.Q636X	1.2	0.3

Age = age at clinical presentation, Symptom = presenting symptom, Size = dimensions on histopathology, MAF = Mutant allelic fraction, MIPs = molecular inversion probe sequencing, ddPCR = droplet digital polymerase chain reaction, ND = not detected, “-”= not performed

Patient characteristics grouped by mutation status are compared in Table 2. The median age of the cohort with an identified mutation was 15.5 years (range 11–46 years) and 6 of 10 were female. There were no significant differences in age at presentation, sex, presenting symptom, or in AVM location, size, radiologic, and histopathologic appearance between patients with and without identified mutations. Differences in clinical characteristics between patients with *KRAS* versus *BRAF* mutations also were not apparent. Although the 2 patients with *BRAF* mutations presented at an older age than the other 14 participants (p = 0.02), our cohort size it too small to determine whether this is clinically meaningful.

**Table 2:** Patient characteristics grouped by mutation status.

		Mutation	No mutation	*P*
Total participants		N = 10	N = 6	
Median age (range) in years		15.5 (11–46)	15.5 (11–20)	0.79
Male sex (%)		4 (40)	3 (50)	1.0
Presenting symptom				0.39
	Headache	6	4	
	Seizure	4	1	
	Hemiplegia	0	1	
Location				0.78
	Occipital	2	2	
	Temporal	2	2	
	Frontal	2	1	
	Parietal	2	0	
	Other	2	1	
Median size, cm^2^ (range)		7.7 (1.8–42)	7.2 (2.6–12)	0.96

## Discussion

We identified *KRAS* or *BRAF* mutations in 63% of the sporadic, intracranial AVM specimens we studied. Nikolaev et al. (2018) first reported *KRAS* mutations in intracranial AVM samples [9]. They examined affected tissue, initially using whole-exome sequencing (WES) and ddPCR, and subsequently using ddPCR alone. In 45/72 (63%) of their intracranial AVM specimens, they identified somatic *KRAS* mutations that are predicted to activate KRAS-mediated signaling. Hong et al. (2019) analyzed 21 brain AVM samples using next generation sequencing with a panel of 422 tumor-related genes (sequencing depth of 1077±298x) and ddPCR. These investigators reported 16 samples contained somatic *KRAS* mutations and 1 sample had a somatic *BRAF* mutation, yielding a mutation detection rate of 81% [10]. Their higher detection rate, compared to our study and Nikolaev et al.’s study [9], likely reflects differences in the criteria employed for calling a variant a “true positive.” For example, we used an MAF > 1% by either MIPseq or ddPCR, and Nikolaev used an MAF > 0.5% by either WES or ddPCR [9]. In contrast, Hong et al. considered some of their ddPCR result positives with a MAF as low as 0.03% [10]. Priemer et al. (2019) tested DNA from formalin-fixed, paraffin-embedded (FFPE) tissue blocks from brain AVMs for 7 common cancer-associated *KRAS* mutations (including p.G12D and p.G12V) and found mutations in 6/21 cases (29%) [11]. Their use of FFPE extracted DNA and an assay that loses sensitivity for a MAF < 1% may account for the lower detection rate in that study [11].

A lack of standardized sampling methods likely explains the variability in reported mutant prevalence. We are the only group that used MIP-seq, while Nikolaev et al. and Hong et al. employed WES and targeted panel sequencing, respectively. MIP-seq was advantageous for our study because it is ideal for screening mutations in a small number of genes (our analysis was limited to genes involving the MAP2K1 signaling pathway). ddPCR, which was used by all three groups, can detect mutant DNA at variable frequencies depending on the specific mutation. For mutations in *KRAS*, detection limits using ddPCR have been reported with MAFs as low as 0.1% [15]. We used a MAF threshold of 1%, however, to label a sample as mutant in order to minimize the false positive rate. A common threshold between study groups would help standardize reporting practices and make intergroup comparisons more meaningful.

Importantly, we provide the second example of a *BRAF* p.V600E mutation in an intracranial AVM and a novel *BRAF* mutation in a second intracranial AVM (p.Q636X). Thus, it seems likely that *BRAF* represents a second locus for which somatic mutations can cause intracranial AVM. Similar to *KRAS*, *BRAF* is a well-known proto-oncogene and the p.V600E mutation is implicated in a number of cancers including melanoma, colorectal carcinoma, and papillary thyroid carcinoma [16–19]. The p.V600E mutation activates B-Raf and downstream signal transduction via the MAP kinase pathway [17,18]. Nonsense mutations in *BRAF* are rarely seen in the COSMIC database, so the mechanism by which the p.Q636X mutation, which is located within the protein’s kinase domain, causes AVM requires further study. This mutation has been detected in ovarian teratoma [20], so it may lead to activating MAP kinase signaling via a novel mechanism.

We did not observe somatic mutations in the other *RAS* family members (*NRAS* or *HRAS*) in our study. Also, although we previously reported that *MAP2K1* mutations are a common cause of extracranial AVM [1], we did not observe *MAP2K1* mutations in any of our intracranial AVM specimens. Interestingly, our detection rate of mutations was similar among our extracranial AVM cohort (16/25; 64%) and our intracranial AVM samples (10/16; 63%). We do not know why somatic mutations have not been found in all brain AVM specimens. Potential explanations include patients having causal mutations that fell below the detection limit of assays we and other investigators employed. Mutations also may reside in genes that have not yet been screened. Alternatively, the surgical sample may have contained minimal AVM nidus containing the mutation. Deep whole genome or whole exome sequencing of specimens without identified mutations should extend the spectrum of genes that are responsible for causing sporadic brain AVMs.

We were unable to identify obvious clinical, radiographic, or histologic features that can be used to predict whether a patient who has a sporadic AVM will have a detectable versus undetectable somatic mutation, or a *KRAS* versus a *BRAF* mutation. Our data are similar to those of Priemer et al. (2019) who also did not find differences in patient characteristics (age, size, and gender) between those with and without *KRAS* mutations [11]. These findings are biased toward resectable AVMs (e.g., brain stem lesions may not be removable). Because patients with and without identifiable mutations were similar, this suggests that the subjects in whom we were unable to find a mutation are more likely to have a *KRAS* or *BRAF* variant compared to a novel mutation in another gene. Our study was limited by having only 16 patients and thus genotype-phenotype associations cannot be ruled-out. Potential trends might prove to be true if larger cohorts of patients were tested (e.g., the 2 patients with *BRAF* mutations were the oldest in the cohort and both were occipital). Future avenues of research would be to continue to test more specimens which might show genotype-phenotype relationships as well as novel mutations in other genes.

Similar to somatic *MAP2K1* mutations in extracranial AVMs [1], we confirmed that somatic *KRAS* mutations in intracranial lesions are also isolated to the endothelial cell [9], suggesting that this cell-type is driving the pathophysiology of AVM. Currently, pharmacotherapy for AVMs does not exist. Development of drugs targeting these activating mutations might prove to prevent the enlargement of both extracranial and intracranial AVMs in the future.

## Supporting information

S1 Table Probe sequences used for gene analysis during molecular inversion probe sequencing. *poorly performing smMIP.
(DOCX)
