# Supplementary material for: Somatic mutations in intracranial arteriovenous malformations
Source: PLoS One. 2019 Dec 31;14(12):e0226852. doi: 10.1371/journal.pone.0226852 (PMC6938308; doi:10.1371/journal.pone.0226852)
Supplement: S1 Table — *poorly performing smMIP. (DOCX) [file pone.0226852.s001.docx]

**S1 Table.** Probe sequences used for gene analysis during molecular inversion probe sequencing.

| Gene | Probe number | Sequence |
| --- | --- | --- |
| NRAS |  |  |
|  | 1 | GTGAGCATATGGTTTCTTGGCATACTTCAGCTTCCCGATATCCGACGGTAGTGTNNNNNNNTTCCTTTTATAGGGTG |
|  | 2 | GGGTTGTATGGGATTGCCATGTGTCTTCAGCTTCCCGATATCCGACGGTAGTGTNNNNNNNGTGTCCGTTGAGCTAG |
|  | 3 | TCAGCATTTGTGCAAGAGTTTGCCTTCAGCTTCCCGATATCCGACGGTAGTGTNNNNNNNCCTATGGTGCTAGTGGG |
|  | 4 | GAAAAACAAGATTAGGCTGGGTACCTTCAGCTTCCCGATATCCGACGGTAGTGTNNNNNNNTCGTGGGCTTGTTTTG |
|  | 5 | GCACTGTACTCTTCTTGTCCAGCTCTTCAGCTTCCCGATATCCGACGGTAGTGTNNNNNNNAACACAAAGATCATCC |
|  | 6 | GGGGGTGTGGAGGGTAAGGGGCTTCAGCTTCCCGATATCCGACGGTAGTGTNNNNNNNGCAAATACACAGAGGAAGC |
|  | 7 | CCAGTGGTAGCCCGCTGACCTGATCTTCAGCTTCCCGATATCCGACGGTAGTGTNNNNNNNGGTTCTTGCTGGTGTG |
| HRAS |  |  |
|  | 1 | GCAGGTGAGGGGGACTCCCTTCAGCTTCCCGATATCCGACGGTAGTGTNNNNNNNATCCCATCCCTCCTTTCCCAGG |
|  | 2 | GCCTTCTACACGTTGGTGCGTGAGCTTCAGCTTCCCGATATCCGACGGTAGTGTNNNNNNNGGGGAAGTGGCTGGTG |
|  | 3 | GTGAGTGCTGCTCCCTGGCTGGGCTTCAGCTTCCCGATATCCGACGGTAGTGTNNNNNNNCTCCCTCACTGCCCTGC |
|  | 4 | GCACCATGGGCACGTCATCCGAGTCTTCAGCTTCCCGATATCCGACGGTAGTGTNNNNNNNTGTGGGGTGGAGAGCT |
|  | 5 | CGGCATCCCCTACATCGAGACCTCCTTCAGCTTCCCGATATCCGACGGTAGTGTNNNNNNNGCTTTCCACCTCTCAG |
|  | 6 | CCTGTCCTCTCTGCGCATGTCCTGCTTCAGCTTCCCGATATCCGACGGTAGTGTNNNNNNNCTTCCTGTGTGTGTTT |
|  | 7 | ACCAGTACAGGTGAACCCCGCTTCAGCTTCCCGATATCCGACGGTAGTGTNNNNNNNTGGGGAGACGTGCCTGTTGG |
|  | 8 | GCCAGGAGGAGTACAGCGCCATGCTTCAGCTTCCCGATATCCGACGGTAGTGTNNNNNNNGAGAGGCTGGCTGTGTG |
|  | 9 | CCACTATAGAGGTGAGCCTGGCGCTTCAGCTTCCCGATATCCGACGGTAGTGTNNNNNNNTGTAGGAGGACCCCGGG |
|  | 10 | GTCCAGGTGCCAGCAGCTGCTGCGCTTCAGCTTCCCGATATCCGACGGTAGTGTNNNNNNNGAGGAGCGATGACGGA |
| KRAS |  |  |
|  | 1 | CGATGGCTTCATGTGTACAGGCTTCAGCTTCCCGATATCCGACGGTAGTGTNNNNNNNCTTTGTCTTTGACTTCTTT |
|  | 2 | CTTAAGGCATACTAGTACAAGTGCTTCAGCTTCCCGATATCCGACGGTAGTGTNNNNNNNGGGTGTTGATGATGCCT |
|  | 3 | GTTCAGCACATTAATTTTGGCAGCTTCAGCTTCCCGATATCCGACGGTAGTGTNNNNNNNCAGAGAGTGGAGGATGC |
|  | 4 | GCAAAGAAGAAAAGACTCCTGGCCTTCAGCTTCCCGATATCCGACGGTAGTGTNNNNNNNTGCACATGGCTTTCCCA |
|  | 5 | AATAAATACAGATCTGTTTTCTGCCTTCAGCTTCCCGATATCCGACGGTAGTGTNNNNNNNCTATGGTCCTAGTAGG |
|  | 6 | GCTCAGGACTTAGCAAGAAGTCTTCAGCTTCCCGATATCCGACGGTAGTGTNNNNNNNGTGTTACTAATGACTGTGC |
|  | 7 | CTGTACTCCTCTTGACCTGCTGTGCTTCAGCTTCCCGATATCCGACGGTAGTGTNNNNNNNACTCCTTAATGTCAGC |
|  | 8 | GGGCTTTCTTTGTGTATTTGCCATCTTCAGCTTCCCGATATCCGACGGTAGTGTNNNNNNNCTGTGTTTCTCCCTTC |
|  | 9 | GCATATTACTGGTGCAGGCTTCAGCTTCCCGATATCCGACGGTAGTGTNNNNNNNGGCCTGCTGAAAATGACTGAAT |
|  | 10 | GCGTAGGCAAGAGTGCCTTGACGCTTCAGCTTCCCGATATCCGACGGTAGTGTNNNNNNNAAAAGGTACTGGTGGAG |
| MAP2K1 |  |  |
|  | 1 | GTAAGTATGGGGCGGGCGGTGCTTCAGCTTCCCGATATCCGACGGTAGTGTNNNNNNNGAGGAAGCGAGAGGTGCTG |
|  | 2 | GCAGAAGGTGGGAGAACTGAAGGACTTCAGCTTCCCGATATCCGACGGTAGTGTNNNNNNNACTTCTCTGGTGACAG |
|  | 3 | GCCAGAAAGGTGAGTTTGCCTTGACTTCAGCTTCCCGATATCCGACGGTAGTGTNNNNNNNCCTTGAGGCCTTTCTT |
|  | 4 | GCCTGGGGACCAGGGTAGAAGGCTTCAGCTTCCCGATATCCGACGGTAGTGTNNNNNNNGAAGATCAGTGAGCTGGG |
|  | 5 | GCGAGATCAGTATCTGCATGGAGCCTTCAGCTTCCCGATATCCGACGGTAGTGTNNNNNNNTCTTCCACCTTTCTCC |
|  | 6 | GCCTTAAGAGTTGGGTGGCTCTGGCTTCAGCTTCCCGATATCCGACGGTAGTGTNNNNNNNAAGGGAGCTGCAGGTT |
|  | 7 | CATTGATAAGTTAATGAGTCGGTCTTCAGCTTCCCGATATCCGACGGTAGTGTNNNNNNNCTGGTCTGGTATTCTCG |
|  | 8 | GTCAGGCCTTTTATTACCTAGAGGCTTCAGCTTCCCGATATCCGACGGTAGTGTNNNNNNNAAGGCTTTTACACGAA |
|  | 9 | GCTTCCTCTTTTTTCTATGTTTTGCTTCAGCTTCCCGATATCCGACGGTAGTGTNNNNNNNTTCTTTTACATTCCCT |
|  | 10 | GCACAAGGTCCTACATGTCGGTCTTCAGCTTCCCGATATCCGACGGTAGTGTNNNNNNNGTTCCCTCCTTTTCTATT |
|  | 11 | CCTGTGGAGCCAGAGTCTTGTGCTTCAGCTTCCCGATATCCGACGGTAGTGTNNNNNNNTTTGGGGTCAGCGGGCAG |
|  | 12 | GGGGTTTCTGGAGGGCTGATTCTTCAGCTTCCCGATATCCGACGGTAGTGTNNNNNNNGCTGATGTTTGGGTGCCAG |
|  | 13 | GTCTGACTGCACAGAGTAATGAGTCTTCAGCTTCCCGATATCCGACGGTAGTGTNNNNNNNTTGGCCTGGGTGGGGT |
|  | 14 | TAACCTAACTTGGACCCCTGGGTCTTCAGCTTCCCGATATCCGACGGTAGTGTNNNNNNNTCTGGAGGAGGGATGGG |
|  | 15 | AATATGAGGCAAGGGGTTGGGCCTTCAGCTTCCCGATATCCGACGGTAGTGTNNNNNNNAAGGAAACCAGGCAGTAC |
|  | 16 | GTTGGCTCCTTGTTCTCTGCTTCAGCTTCCCGATATCCGACGGTAGTGTNNNNNNNGAGGGGTGGGATGGGGAGAGG |
|  | 17 | GCTGTTGCCTGGCACTGGTTCTCTTCAGCTTCCCGATATCCGACGGTAGTGTNNNNNNNGATGACTGACAGAGAAGA |
|  | 18 | GCTGGCGTCTAAGTGTTTGGGAAGCTTCAGCTTCCCGATATCCGACGGTAGTGTNNNNNNNTTAACACCACGTCCTC |
|  | 19 | GCTGGGCTGGTTAAGGCCGATGGTCTTCAGCTTCCCGATATCCGACGGTAGTGTNNNNNNNGTCACAGGTGAAATGC |
| BRAF |  |  |
|  | 1* | GCACTGCGGTGAATTTTTGGCAATCTTCAGCTTCCCGATATCCGACGGTAGTGTNNNNNNNCTCTCTCACTCATTTG |
|  | 2* | CCCTCCTTGAATCGGGCTGGCTTCAGCTTCCCGATATCCGACGGTAGTGTNNNNNNNCTGTTTTTTCTTTTTCTTTC |
|  | 3* | GCTTCATGCTATCCAAAAGAACAGCTTCAGCTTCCCGATATCCGACGGTAGTGTNNNNNNNATGGTGGGACGAGGAT |
|  | 4 | AGACCACTCTTTCCCCAAGTAACTTCAGCTTCCCGATATCCGACGGTAGTGTNNNNNNNCCCTTTTTTTTCTCTCTC |
|  | 5 | GCTTTTACCATTAAAACACCTGTCCTTCAGCTTCCCGATATCCGACGGTAGTGTNNNNNNNTCTGACTGAAAGCTGT |
|  | 6 | GCATTCTGATGACTTCTGGTGCCTCTTCAGCTTCCCGATATCCGACGGTAGTGTNNNNNNNAACACCAAGACGTGGT |
|  | 7 | AAATTTTTGGCCCTGAGATGCTGCTTCAGCTTCCCGATATCCGACGGTAGTGTNNNNNNNATGAAGACCTCACAGTA |
|  | 8 | GTTGTCTGGATCCATTTTGTGGCTTCAGCTTCCCGATATCCGACGGTAGTGTNNNNNNNAGGAAAATGAGATCTACT |
|  | 9 | GTAGGGCTAAAGGACTCTGGCCTTCAGCTTCCCGATATCCGACGGTAGTGTNNNNNNNCAAAATGCAGAAGAAAAAG |
|  | 10 | GCCAGTTGTGGCTTTGTGGCTTCAGCTTCCCGATATCCGACGGTAGTGTNNNNNNNAGACATTTAACGAATGGAACT |
|  | 11 | GTTACCCAGTGGTGTGAGGGCTCCTTCAGCTTCCCGATATCCGACGGTAGTGTNNNNNNNCTTTGGTTCTCTTTTGT |
|  | 12 | GCAATTGTTACTCCAAGTGTCATTCTTCAGCTTCCCGATATCCGACGGTAGTGTNNNNNNNAAATAACTTCTTTCTC |
|  | 13 | CAAAGGAAATATTCACTGTTCGCCTTCAGCTTCCCGATATCCGACGGTAGTGTNNNNNNNGTGGGACAAAGAATTGG |
|  | 14 | GCCAAACAGAAAAAGAAAACCCTTCAGCTTCCCGATATCCGACGGTAGTGTNNNNNNNTCCCTTGTAGACTGTTCCA |
|  | 15 | GTAATGAGGCAGGGGGGGTAGCTTCAGCTTCCCGATATCCGACGGTAGTGTNNNNNNNAAGAAGAAAGAATTCAGAG |
|  | 16 | ATCAGGGGTAGAAGGTTGGGGGACTTCAGCTTCCCGATATCCGACGGTAGTGTNNNNNNNGGAGATTTCTGTAAGGC |
|  | 17 | GGAAACTGCAATTGCTTTGCTGCCTTCAGCTTCCCGATATCCGACGGTAGTGTNNNNNNNGTGTTATCGCTACTCTC |
|  | 18 | GGAATGGATTTTGAAGGAGACGGCTTCAGCTTCCCGATATCCGACGGTAGTGTNNNNNNNAGCAATGCTGGATACTT |
|  | 19 | GTCATTGGAAGATAAGATTCAGAGCTTCAGCTTCCCGATATCCGACGGTAGTGTNNNNNNNTTGGGAGCTGATGAGG |
|  | 20 | GCTCTTGGGCGACATGCTACTTGCTTCAGCTTCCCGATATCCGACGGTAGTGTNNNNNNNGTTTGCTGTTTGTCTCC |
|  | 21 | TTCCATTCTCAGAAACAACTGCTTCAGCTTCCCGATATCCGACGGTAGTGTNNNNNNNCCTGTGGTATTGGGTGGTG |
|  | 22 | GGAAAAGCAGCTTTCGACAAAAGTCTTCAGCTTCCCGATATCCGACGGTAGTGTNNNNNNNAGTGTAAAATGGTAGG |
|  | 23 | GGTTTCCGCTGTCAAACATGTGGTCTTCAGCTTCCCGATATCCGACGGTAGTGTNNNNNNNCAAGAGAGTAGATACG |
|  | 24 | CTTCTCCAGTAAGCCAGGCTTCAGCTTCCCGATATCCGACGGTAGTGTNNNNNNNACTCATCCATATTTCACATTCC |
|  | 25 | GAAGTGTTGGAGAATGTTCCACCTTCAGCTTCCCGATATCCGACGGTAGTGTNNNNNNNCATATCTTCAGCATGAGG |
|  | 26 | GTATGGTTTGTATGTGACGCTTCAGCTTCCCGATATCCGACGGTAGTGTNNNNNNNCCCTTTACCTCTTATCAATAT |
|  | 27 | GATGAAGGTAGCACTGAAAGGCCTTCAGCTTCCCGATATCCGACGGTAGTGTNNNNNNNGAAACAGCAAAATGGTGA |
|  | 28 | CTGTTGTTCTCTTTGTTGGAGTGCCTTCAGCTTCCCGATATCCGACGGTAGTGTNNNNNNNCATCTGTGGGATTTTG |
|  | 29 | GCTCTGCATCAATGGATACCGCTTCAGCTTCCCGATATCCGACGGTAGTGTNNNNNNNGGACAAAGTCCGGATTGAA |
|  | 30 | TCTTGATTATTTTAGGAGGTGGGCTTCAGCTTCCCGATATCCGACGGTAGTGTNNNNNNNATATAGAGGCCCTATTG |
|  | 31 | CATAGACAACTACATCACAGCTTCAGCTTCCCGATATCCGACGGTAGTGTNNNNNNNGCTCCCCACCAAATTTGTCC |
|  | 32 | GACAAATTTGGTGGGGAGCCTTCAGCTTCCCGATATCCGACGGTAGTGTNNNNNNNCTGTGATGTAGTTGTCTATGT |
|  | 33 | GGAGGCGGAGGCGGAGGCGGAGGACTTCAGCTTCCCGATATCCGACGGTAGTGTNNNNNNNTTGAACAGAGCCTGGC |
|  | 34 | CGGAGGAGGTGAGTGCTGGCGCTTCAGCTTCCCGATATCCGACGGTAGTGTNNNNNNNGGTTATAAGATGGCGGCGC |
|  | 35 | GCTCGGCGGCTGGCTGGTGTTTATCTTCAGCTTCCCGATATCCGACGGTAGTGTNNNNNNNGGGCCAGGCTCTGTTC |

*poorly performing smMIP
